# Draft Genome Sequence of Cold-Tolerant Kurthia gibsonii B83, Isolated from Spinach Leaf

**DOI:** 10.1128/MRA.01480-18

**Published:** 2019-03-14

**Authors:** Bidhan Chandra Mukhopadhyay, Suranjita Mitra, Tawsif Ahmed Kazi, Sukhendu Mandal, Swadesh Ranjan Biswas

**Affiliations:** a Department of Botany, Visva-Bharati, Santiniketan, West Bengal, India; b Department of Microbiology, University of Calcutta, Kolkata, India; University of Arizona

## Abstract

Limited information is available on the whole-genome sequences of *Kurthia* spp. Here, we report, for the first time, the draft genome sequence of Kurthia gibsonii designated as strain B83. The strain was isolated from spinach (Spinacia oleracea L.) leaf. The genome was sequenced on the Illumina NextSeq 500 platform.

## Abstract (precis)

Limited information is available on the whole-genome sequences of *Kurthia* spp. Here, we report, for the first time, the draft genome sequence of Kurthia gibsonii designated as strain B83.

## ANNOUNCEMENT

Members of the genus *Kurthia* are Gram-positive bacteria that belong to the family *Planococcaceae*, order *Bacillales*, class *Bacilli*, and phylum *Firmicutes* and comprise a few species, namely, K. zopfii, K. gibsonii, K. sibirica, K. massiliensis, K. huakuii, and K. senegalensis. The members of the *Kurthia* genus are found in decomposing organic material, meats, meat products, and milk. Information on the genome sequences of *Kurthia* spp. is limited. We have isolated a strain of Kurthia gibsonii from spinach leaf collected from an agricultural field in Sahapur, Bankura, West Bengal, India. The fresh-cut leaf was inoculated into tryptone, glucose, and yeast extract (TGE) medium (all at 1% and pH 6.5; HiMedia) (1) for enrichment of bacteria. After overnight incubation at 37°C, the sample was serially diluted and plated on TGE agar as described previously (2). The colony had a bird's feather appearance and was repeatedly streaked on TGE agar to obtain a pure culture. It grew at temperatures from 10°C to 45°C, similar to those obtained previously (3). Genomic DNA from a single colony of the pure culture was isolated for PCR amplification of the 16S rRNA gene with 27F (5′-AGAGTTTGATCATGGCTC-3′) and 1327R (5′-CTAGCGATTCCGACTTCA-3′) bacterium-specific universal primers, as described previously (2). The BLAST analysis revealed 99% to 100% similarity with different strains (99% query coverage) of Kurthia gibsonii only in the GenBank database. Hence, the isolate was identified as K. gibsonii and designated K. gibsonii B83. So far, limited information is available on the sequenced genomes of *Kurthia* spp. This prompted us to sequence the genome of Kurthia gibsonii B83.

A single colony of K. gibsonii B83 was grown overnight in TGE medium at 37°C, and then the cells were lysed with lysozyme (10 mg/ml) followed by purification of genomic DNA using the Qiagen Genomic-tip 100/G and the Qiagen genomic DNA buffer set. An Illumina TruSeq nano DNA library kit was used to prepare the paired-end libraries, and the genome was sequenced with 2 × 150-bp chemistry on the Illumina NextSeq 500 platform. The sequenced raw data were processed to obtain high-quality clean reads using Trimmomatic v0.35 (quality threshold [QV], <20 Phred score) (4) and quality checked using FastQC (5). The reads were *de novo* assembled using Velvet 1.2.10 with default parameters (6), and, scaffold construction was done subsequently using SSPACE-Standard v3.0 with default parameters (7). Finally, 47 scaffolds (2,947,201 bp; G+C content of 36.4%; *N*_50_ value, 140,789; 387.84× coverage) were generated from 3,869,243 paired-end reads. The genome was functionally annotated using NCBI Prokaryotic Genome Annotation Pipeline (8). Totals of 2,858 coding sequences, 80 RNAs, and 1 clustered regularly interspaced short palindromic repeat (CRISPR) array were identified. Genome annotation of K. gibsonii B83 revealed the presence of a number of stress tolerance genes. Further experimental work will be required to gain insight into the cold adaptation mechanism of this strain.

### Data availability.

This whole-genome shotgun project has been deposited at DDBJ/ENA/GenBank under the accession number QZNE00000000. The version described in this paper is the first version, QZNE01000000. Raw sequencing data have been submitted to the NCBI Sequence Read Archive database under the accession number PRJNA492835.
